# Application of Hybrid Electrobaromembrane Process for Selective Recovery of Lithium from Cobalt- and Nickel-Containing Leaching Solutions

**DOI:** 10.3390/membranes13050509

**Published:** 2023-05-11

**Authors:** Dmitrii Butylskii, Vasiliy Troitskiy, Daria Chuprynina, Lasâad Dammak, Christian Larchet, Victor Nikonenko

**Affiliations:** 1 Membrane Institute, Kuban State University, 149 Stavropolskaya St., 350040 Krasnodar, Russia; 2 Department of Analytical Chemistry, Kuban State University, 149 Stavropolskaya St., 350040 Krasnodar, Russia; 3 CNRS, ICMPE, UMR 7182, Université Paris-Est Créteil, 2 Rue Henri Dunant, 94320 Thiais, France

**Keywords:** lithium extraction, spent lithium-ion battery, ion separation, electrobaromembrane separation, countercurrent electromigration

## Abstract

New processes for recycling valuable materials from used lithium-ion batteries (LIBs) need to be developed. This is critical to both meeting growing global demand and mitigating the electronic waste crisis. In contrast to the use of reagent-based processes, this work shows the results of testing a hybrid electrobaromembrane (EBM) method for the selective separation of Li^+^ and Co^2+^ ions. Separation is carried out using a track-etched membrane with a pore diameter of 35 nm, which can create conditions for separation if an electric field and an oppositely directed pressure field are applied simultaneously. It is shown that the efficiency of ion separation for a lithium/cobalt pair can be very high due to the possibility of directing the fluxes of separated ions to opposite sides. The flux of lithium through the membrane is about 0.3 mol/(m^2^ × h). The presence of coexisting nickel ions in the feed solution does not affect the flux of lithium. It is shown that the EBM separation conditions can be chosen so that only lithium is extracted from the feed solution, while cobalt and nickel remain in it.

## 1. Introduction

The development of new approaches for the extraction of valuable components from aqueous solutions is an important task. For the extraction of alkali, alkaline-earth and transition metal compounds on an industrial scale, pyrometallurgy, reagent-based methods of hydrometallurgy, sorption, electrochemical reduction, etc., having high efficiency are often used. Multistage ion separation processes are used in industry for the production of lithium, cobalt, nickel, zinc, titanium, etc., with high purity [1,2,3]. All these processes are well developed when treating natural sources. However, the valuable metals listed above can also be obtained from secondary sources (e-waste, sludge, ash, tailing, etc.) [4,5,6]. The problem is acute when using, for example, lithium-ion batteries (LIBs). Tests of lithium materials newly obtained from spent LIBs have shown that they are not inferior in performance to materials made from lithium obtained from primary sources (natural brines and minerals) [7,8,9], but all of them cannot be called environmentally friendly.

The authors of review papers [1,2,3] note that the extraction of lithium and no-less-valuable cobalt from leachates of spent LIBs has clear advantages over their extraction from natural sources. First, the concentration of lithium and cobalt in leachates is usually quite high. Second, the composition of leachates is more predictable and less varied. Third, leachates do not contain multiply charged ions such as Ca^2+^ and Mg^2+^, which have low value and which make it difficult to extract lithium from natural solutions. In addition, a significant advantage is the reduction of the environmental burden due to the neutralization of spent LIBs as hazardous waste [10].

However, it is difficult to organize a LIB processing plant. This is mainly due to the need to sort different types of batteries and the complexity of their disassembly, as well as the high reactivity of materials in spent LIBs, which can ignite and explode on contact with air [11]. For these reasons, the reuse of spent LIBs is insignificant. For example, in Australia (world leader in lithium mining) in 2017–2018, only 6% of LIBs that were out of service during this period were recycled [12]. The profit from the recycling of different types of spent LIBs also differs. Bhandari et al. [13] note that the processing of NMC batteries (the cathode is obtained from a mixture of lithium, nickel, manganese and cobalt) is more economically attractive than the recycling of cheaper LFP batteries (lithium iron phosphate cathode). This is due to the ability to extract valuable nickel and cobalt from leachates of NMC batteries in addition to lithium, as in the case of LFP.

In industry, the processing of spent LIBs is carried out using three traditional methods: pyrometallurgy, hydrometallurgy, or a combination of these. Pyrometallurgical processing does not allow the extraction of lithium from spent LIBs; it goes to the slag [10]. The target component is valuable Co and other metals [14]. Lithium can be recovered from spent LIBs using hydrometallurgical processes. According to the latest estimates [13], one ton of spent NMC811-type LIBs can bring a profit from the sale of materials of at least USD 6500 (in 2021). More than 50% of the profit will come from recovered lithium.

Membrane methods for separating the components of spent LIBs are still under development [1]. Selective electrodialysis (SED) is most commonly used to extract Li^+^ ions from LIB leachates [15,16,17,18]. The SED technology differs from conventional electrodialysis in the use of multiple units of at least one special-grade ion-exchange membrane (cell pair). These membranes are commercially available [1,2,3]. They pass singly charged ions well and reject multiply charged ones. This makes it possible to efficiently separate Li^+^ ions from Co^2+^, Ni^2+^ and Mn^2+^ [18].

Another attractive membrane technology is the hybrid electrobaromembrane (EBM) method [1]. Unlike electrodialysis, EBM separation uses nonselective porous membranes. Separated ions of the same charge sign move in an electric field through the pores of this membrane to the corresponding electrode, while a commensurate counter convective flow is created in the pores. The selectivity of separation is achieved due to the difference in the mobility of the competing ions [19,20,21,22].

In recent studies on EBM devices, impressive results have been achieved in the separation of Li^+^/K^+^ ions when using feed solutions imitating natural waters [23,24,25]. It was shown that the ion separation coefficient for the Li^+^/K^+^ ions can be as high as 59 [23,26] or even 150 [24,25]. For the Li^+^/Na^+^ pair, the selective permeability coefficient is somewhat lower, reaching 30 [24]. However, the EBM method and porous membranes have not yet been tested in the recovery of lithium from secondary sources represented by leachates or liquors of spent LIBs.

In this regard, the purpose of this study is to expand the scope of the EBM method, as well as to study the possibility of using nanoporous membranes that do not have selectivity for a certain type of ions but ensure their selective separation. The paper presents the results of testing a hybrid EBM method for separating Li^+^ and Co^2+^ ions, as well as Li^+^, Co^2+^ and Ni^2+^ ions contained in leachates of spent LIBs. The efficiency of the EBM method is analyzed, and the parameters of ion separation are compared with other membrane methods.

## 2. Materials and Methods

Two types of feed solutions were used. A mixture of lithium and cobalt sulfates was used to determine the optimal separation parameters, and then nickel sulfate was added to this mixture to test the possibility of separating lithium from a more complex mixture. The main characteristics of the feed solution components that affect the efficiency of EBM separation are presented in Table 1.

A mixture of 0.05 M Li_2_SO_4_ and 0.05 M CoSO_4_ was used as the first type of feed solution (pH = 4.2–4.4) and a mixture of 0.05 M Li_2_SO_4_, 0.025 M CoSO_4_ and 0.025 M NiSO_4_ was used as the second type of feed solution (pH = 5.2). The concentrations of the components were within wide limits, which are typical for solutions of spent LIB leachates.

In this work, a track-etched membrane (designated as TEM #811) was used as a nanoporous membrane. It was produced from a polyethylene terephthalate (PET) film at the Joint Institute for Nuclear Research (Dubna, Russia). The properties of the TEM are described in Table 2.

**Figure 1:**
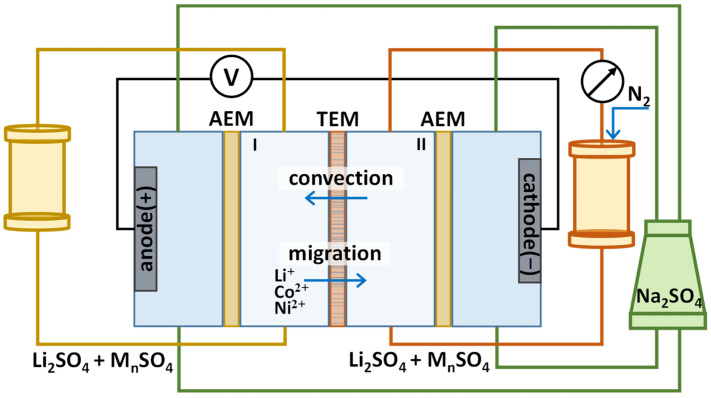
Schematic diagram of the setup for studying the parameters of the selective separation of Li^+^/Co^2+^ and Li^+^/Co^2+^/Ni^2+^ cations using the hybrid electrobaromembrane method.

On the left- and right-hand sides the TEM is surrounded by auxiliary anion-exchange (AEM) MA-41 heterogeneous membranes (JCC Shchekinoazot, Pervomayskiy, Russia) to form flow chambers. Solutions of the same composition and volume (0.15 L) were pumped through the left-hand (I) and right-hand (II) chambers, separated by a porous membrane, at the same flow rate (5.4 L/h) (Figure 1). A 0.1 M Na_2_SO_4_ solution was pumped through the electrode chambers (4 L). Separation experiments at given parameters were repeated at least four times. The duration of each experiment was 8 h; this amount of time was needed to measure the fluxes of competing ions at a given electric current and a pressure drop in a steady state of the system. A convective flow directed from chamber II to chamber I was created opposite to the electromigration of the competitive cations. This was achieved by increasing the pressure of the solution in the circuit passing through chamber II using an automatic nitrogen dosing system.

Samples of the solutions from chambers I and II were taken at the beginning and the end of the separation process to determine the concentration of Li^+^-ions using a Dionex ICS-3000 ion-chromatograph with a conductometric detector (Dionex, Sunnyvale, CA, USA). The concentration of cobalt and nickel (if any) was determined using direct spectrophotometric analysis with a UV-1800 TM ECOVIEW (Shanghai Mapada Instruments Co., Shanghai, China) instrument.

## 3. Results

**Figure 2:**
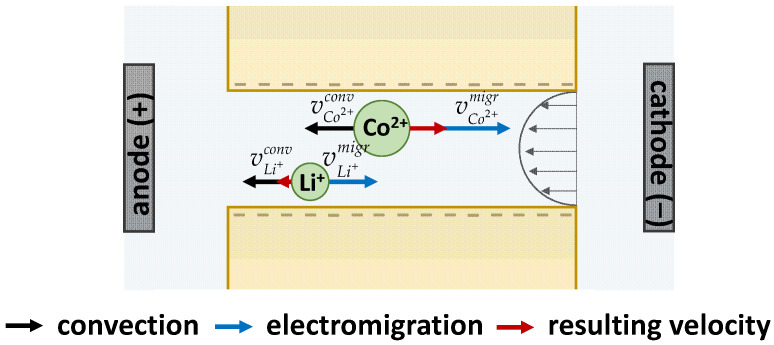
Scheme of ion velocities in the pore of a track-etched membrane. The velocity of electromigration (blue arrows) is proportional to the ion mobility and electric field; the velocity of convective transfer (black arrows) depends only on the pressure drop and is the same for both ions. The resulting velocities (red arrows) can be directed to different sides.

Let us consider the mechanism of ion separation by the hybrid electrobaromembrane method in more detail (Figure 2). When only an external electric field is applied, the separated Li^+^ and Co^2+^ ions migrate through the pores of the TEM to the negative cathode and their velocities are proportional to their mobility *u_k_* (or diffusion coefficient *D_k_*, multiplied by the charge numbers *z_k_*): $v_{k}^{migr}=u_{k}E=\left(D_{k}z_{k}F/RT\right)E$, where *E* is the electric field strength. Under the action of one driving force (electric field), Li^+^ and Co^2+^ ions are freely transferred through the wide pores of the membrane.

When a pressure field is applied to the system along with the electric field, a convective flow is created in the pores of the TEM. The convective flow is opposite to the electromigration flow of separated ions. The velocity of convection is the same for both separated ions. For efficient separation, it is necessary to choose the ratio of the rates of convection and electromigration so that the resulting rate of the least mobile ion (here, these are Li^+^ ions) tends to zero. It is also possible to choose conditions such that the ions to be separated move in different directions, as shown in Figure 2.

### 3.1. Theory of EBM Separation

Separation of ions by the EBM method occurs under the action of the two external forces mentioned above. Additionally, a concentration difference may appear when the solutions are different on both sides of the membrane. Therefore, when calculating the fluxes of competing counterions (*k* = 1, 2), it is necessary to take into account electromigration, convection and diffusion contributions [23]:

(1) $$j_{1}=j_{1}^{migr}+j_{1}^{dif}+j_{1}^{conv}=\frac{i{\tilde{t}}_{1}}{z_{1}F}+c_{1}v^{conv}\gamma$$

(2) $$j_{2}=j_{2}^{migr}+j_{2}^{dif}+j_{2}^{conv}=\frac{i{\tilde{t}}_{2}}{z_{2}F}+c_{2}v^{conv}\gamma$$

where *i* is the current density and *j_k_*, ${\tilde{t}}_{k}$, *c_k_* and *z_k_* are the flux density, effective transport number, concentration and charge number of cation *k*; $v^{conv}$ is the convective velocity; and *γ* is the surface porosity (the fraction of the membrane surface occupied by the pore openings).

The contribution of diffusion is taken into account implicitly through the value of the effective transport number. The ${\tilde{t}}_{k}$ value characterizes the fraction of electric charge carried by ion *k* under the action of electric current and diffusion (if any). It is easy to see that in Equations (1) and (2), only the ${\tilde{t}}_{k}$ and $v^{conv}$ values are unknown. However, assuming that the convective velocity depends only on the pressure drop and is independent of the given current, this can be estimated by the Hagen-Poiseuille equation:

(3) $$v_{}^{conv}=\frac{1}{32}\frac{\Delta pd^{2}}{\eta L}$$

where ∆*p* is the pressure difference between chamber II and chamber I, *d* and *L* are the diameter and length of a pore, and *η* is the liquid viscosity. 

Now only ${\tilde{t}}_{k}$ is unknown, and can be determined by the fit between the experimental and calculation results. At the same time, taking into account a relatively large pore diameter, ${\tilde{t}}_{k}$ cannot be much greater than the (electromigration) transport numbers in a free solution ${\tilde{t}}_{k}$:

(4) $${\tilde{t}}_{k}\approx t_{k}=\frac{z_{k}^{2}D_{k}c_{k}}{\sum_{j=1,2,3}z_{j}^{2}D_{j}c_{j}}$$

Due to the different mobility of competing ions in an electric field, it is possible to choose values of the set current and pressure drop such that the flux of one of them through the track-etched membrane tends to zero [1,23,24]. Let us suppose that $j_{2}=0$; Equation (2) can be used to express the value of the convective velocity:

(5) $$v^{conv}=\frac{i{\tilde{t}}_{2}}{z_{2}c_{2}F\gamma }$$

Substituting Equation (3) in Equation (1), taking into account that the convective flow is opposed to the electromigration flow, the following expression can be obtained:

(6) $$j_{1}=\frac{i}{F}\left(\frac{{\tilde{t}}_{1}}{z_{1}}-\frac{{\tilde{t}}_{2}}{z_{2}}\frac{c_{1}}{c_{2}}\right)$$

If Equation (4) is taken into account, expression (6) can be rewritten in the following form:

(7) $$j_{1}\approx \frac{i{\tilde{t}}_{1}}{z_{1}F}\left(1-\frac{z_{2}D_{2}}{z_{1}D_{1}}\right)$$

Conducting a brief analysis of Equation (7), it should be noted that the flux density of the ions, which has the highest mobility in an electric field, increases as the $z_{2}D_{2}/z_{1}D_{1}$ ratio decreases. If $z_{2}D_{2}$ is close to $z_{1}D_{1}$, the competing ions cannot be separated.

### 3.2. Separation of Li^+^/Co^2+^-Ions

**Figure 3:**
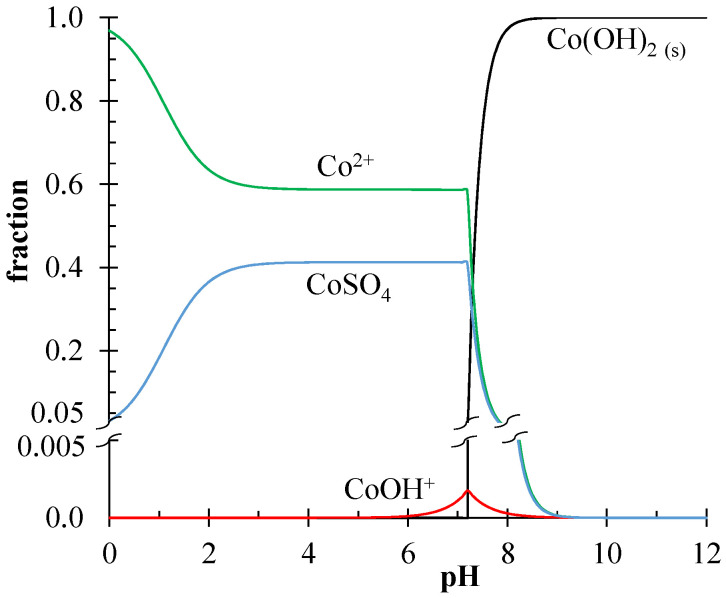
Equilibrium diagram of cobalt compounds in an aqueous solution obtained using the Medusa/Hydra software package [31].

Lithium and cobalt have different electromigration velocities $v_{k}^{migr}$, since this velocity is proportional to the electrical mobility of the ions. Cobalt ions are more mobile in an electric field than lithium ions. The $z_{2}D_{2}/z_{1}D_{1}$ ratio for the Li^+^/Co^2+^ pair is about 0.71. This means that, from a theoretical point of view, the separation of these ions by the EBM method is possible. However, it is known that cobalt sulfate exists in neutral and acid aqueous solutions in two forms: Co^2+^ and CoSO_4_ (Figure 3). According to the Medusa/Hydra software, at the pH value of the feed solution (4.2–4.4), the fraction of Co^2+^ ions is 0.6. For calculations, the values of the equilibrium constants from the Hydra built-in database were used [31].

Taking into account the proportion of Co^2+^ ions in the solutions pumped through chamber I and chamber II, the transport numbers of lithium and cobalt ions in free solution in Equation (4) are as follows: $t_{Li^{+}}$ = 0.17 and $t_{Co^{2+}}$ = 0.14, while $t_{SO_{4}^{2-}}$ = 0.69. Due to the relatively large pore size (*d*_av_ = 35 ± 3 nm), the transport numbers ${\tilde{t}}_{k}$ of separated ions in a nanoporous membrane should not differ significantly from their transport numbers in solution. This means that the flux of cobalt ions through the membrane determined by electromigration should be lower than the lithium flux, despite the cobalt ions having higher mobility in an electric field.

To determine the experimental flux of cation *k* under separation (*k* = Li^+^ or Co^2+^), it is necessary to measure the quasi-stationary rate of change of the cation concentration $dc_{k}/dt$ in chamber I or chamber II:

(8) $$j_{k}=\frac{V}{s}\frac{dc_{k}}{dt}$$

where *V* is the volume of solution in chamber I or chamber II, *s* is the membrane surface area, and *t* is the duration of an experiment.

The efficiency of ion separation is characterized by the ion separation coefficient $S_{Li^{+}/Co^{2+}}$(also called the permselectivity coefficient between two counterions) [32,33]:

(9) $$S_{Li^{+}/Co^{2+}}=\frac{j_{Li^{+}}/j_{Co^{2+}}}{c_{Li^{+}}^{0}/c_{Co^{2+}}^{0}}=\frac{\Delta c_{Li^{+}}^{}/\Delta c_{Co^{2+}}^{}}{c_{Li^{+}}^{0}/c_{Co^{2+}}^{0}}$$

where $j_{Li^{+}}$ and $j_{Co^{2+}}$ are the flux densities of Li^+^ and Co^2+^ through the membrane, respectively; $c_{Li^{+}}^{0}$ and $c_{Co^{2+}}^{0}$ are the concentrations of these ions in chamber I or chamber II (in our case, the solutions in these chambers are the same); and $\Delta c_{Li^{+}}^{}$ and $\Delta c_{Co^{2+}}^{}$ are the changes in concentrations of these ions in chamber I or chamber II. Note that when calculating the value of $S_{Li^{+}/Co^{2+}}$, the total concentration of cobalt was used, and this can exist in two forms: as a doubly charged cation and in the composition of cobalt sulfate. The method of cobalt concentration measurement (see experimental section) also determines its total concentration. A similar approach has been previously used in other studies [34,35].

**Figure 4:**
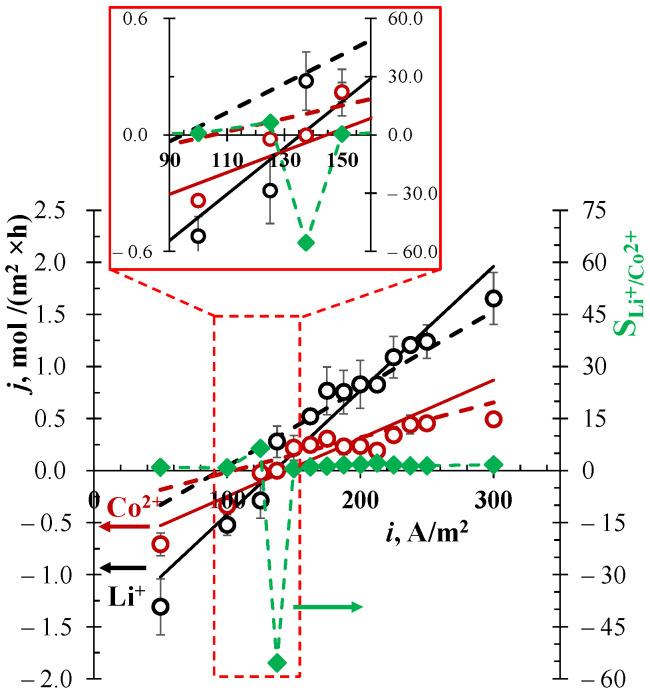
Flux densities of Li^+^ (black circles) and Co^2+^ ions (dark red circles) through the TEM #811 membrane, and the ion separation coefficient $S_{Li^{+}/Co^{2+}}$ (diamonds with dashed green line) vs. the current density for the EBM system at a constant pressure drop of 0.3 bar. Experimental data are shown by markers; lines are calculated using Equations (1)–(3), taking into account *d* = 32 nm, ${\tilde{t}}_{Li^{+}}$ = 0.32 and ${\tilde{t}}_{Co_{}^{2+}}$ = 0.15 (black and dark red solid lines) and *d* = 26 nm, with ${\tilde{t}}_{Li^{+}}$ = 0.20 and ${\tilde{t}}_{Co_{}^{2+}}$ = 0.09 (black and dark red dashed lines) as fitting parameters.

Figure 4 shows the results of the separation of Li^+^ and Co^2+^ ions using the EBM method, as well as the results of the calculation using Equations (1)–(3). The experiments were carried out at the constant pressure drop of 0.3 bar between chambers II and I, since this value provided optimal separation without significant overflow of solution from the chamber under pressure II to the adjacent chamber I.

When a low current value (50 A/m^2^) is set in the system, the experimental values of the fluxes of both separated ions are negative at ∆*p* = 0.3 bar. This means that the process is controlled by the convection. Since both separated ions experience the same effect of convection, one should not expect high separation selectivity with these parameters. When current density is in the range 125–137.5 A/m^2^, the flux of cobalt ions through the membrane tends to zero. In this case, the flux of lithium ions is significant, and is determined either by convection (at 125 A/m^2^) or migration (at 137.5 A/m^2^). This leads to an increase in selectivity, with a value ranging from 8 up to –55. It is important to note that at 125 A/m^2^ the fluxes of lithium and cobalt are negative and codirectional. At 137.5 A/m^2^, the lithium flux becomes positive, while the cobalt flux remains negative. Taking into account the fact that only lithium ions can leave the feed solution, i.e., the outgoing fluxes of the competing ions are zero, the separation coefficient should formally be set to infinity. However, since the feed solution and receiving solution in experiments were identical, $S_{Li^{+}/Co^{2+}}$ cannot be evaluated as infinity. Hereafter, when the fluxes of lithium and competing ions have opposite signs, “not available for calculation” will be written and abbreviated as “n/a”.

This feature makes it possible to effectively separate Li^+^ and Co^2+^ ions despite the obtained value of the ion separation coefficient. With a further increase in current at ∆*p* = 0.3 bar, the ion separation coefficient does not differ much from 1.

The values of the transport numbers ${\tilde{t}}_{k}$ of competing cations in a nanoporous membrane were determined by the best fit between the experiment and the theory. When *d* = 32 nm, the fitted values were 0.32 and 0.15 for lithium and cobalt, respectively. The difference from their transport numbers in solution ($t_{Li^{+}}$ = 0.17 and $t_{Co^{2+}}$ = 0.14) is probably due to the error in determining the average pore diameter (35 ± 3 nm according to SEM results). It was recently found that a loose cation-conductive intermediate gel layer between the pore solution and the nonconductive membrane bulk material can form in the pores of TEMs during track etching [36,37]. Some of the cations driven by the electric field pass through this loose layer, which leads to an increase in their transport numbers.

On the other hand, the formation of a loose layer can lead to narrowing in the middle part of the pores. Indeed, if the hydraulic permeability of TEM #811 is taken into account, then using the Hagen-Poiseuille Equation (3), an average pore size of 28 ± 2 nm can be obtained [23,36]. Figure 4 shows good agreement between the theoretical dependence (dashed lines) and positive values of the fluxes corresponding to the dominant migration. Here, the average diameter calculated from the hydraulic permeability (26 nm) and the values of the fitted transport numbers becomes closer to those in free solution (${\tilde{t}}_{Li^{+}}$= 0.20 and ${\tilde{t}}_{Co^{2+}}$ = 0.09 were used).

Since at the beginning of the experiment, the solutions on both sides of the TEM were the same, and the composition of these solutions changed only slightly (by less than 20%) during the experiment, the contribution of diffusion to the ion transport can be ignored. Therefore, the effective transport numbers in Equations (1) and (2) should be close to the electromigration transport numbers.

In addition, the presented theoretical analysis does not take into account the interaction of the separated cations with the hydroxyl and carboxyl groups of the polyethylene terephthalate TEM [38]. The negative charge of the pore walls will attract the lithium and cobalt ions inside the pore (mainly in the electrical double layer on the pore walls). As a result, the cation transport numbers in the pore should be slightly greater than in the free solution. Moreover, a fixed charge will affect doubly charged cobalt ions to a greater extent than lithium ions [39].

### 3.3. Separation of Li^+^/Co^2+^/Ni^2+^-Ions

In addition to lithium and cobalt, the leachates of spent LIBs also contain nickel and manganese ions, which are less valuable [1,2,3,18]. The literature analysis allows us to conclude that the presence of coexisting ions in the feed solution significantly affects the efficiency of separation by electrodialysis [15,18,40]. For example, Ji et al. [40] showed that the presence of Na^+^, K^+^ and Ca^2+^ ions in natural water (used as a feed solution) negatively affects the efficiency of separation of Mg^2+^ and Li^+^ by selective electrodialysis. With an increase in the $c_{Na^{+}}/c_{Li^{+}}$ ratio in the feed solution from 1 to 20, $S_{Mg^{2+}/Li^{+}}$ decreases from 8.7 to 1.8. The negative effect is associated with a decrease in the flux of Li^+^ ions through the membrane in the presence of an excess of Na^+^ ions. The presence of Ni^2+^ and Mn^2+^ ions in leachates as a feed solution similarly affects the flux of Co^2+^ ions through the membrane [15,18].

To evaluate the effect of coexisting Ni^2+^ ions on the separation efficiency of Li^+^ and Co^2+^ ions through the TEM #811 membrane, optimal parameters selected from the dependence in Figure 4 (∆*p* = 0.3 bar; *i* = 137.5 A/m^2^) were used. The concentration of cobalt ions was reduced by half (usually the ratio of Co^2+^ and Ni^2+^ ions in leachates is approximately 1:1 [16,18]), due to which the ionic strength of the solution did not change. The composition of the feed solution was as follows: 0.05 M Li_2_SO_4_, 0.025 M CoSO_4_ and 0.025 M NiSO_4_ (pH = 5.2). Table 3 shows the results of the separation of Li^+^/Co^2+^-ions by the EBM method in the presence of Ni^2+^ ions.

Under selected conditions, in the presence of Ni^2+^ ions, the selectivity of Li^+^/Co^2+^-ion separation is significantly reduced ($S_{Li^{+}/Co^{2+}}$= 4). The flux of lithium ions through the membrane remains the same, within experimental error, and the flux of cobalt ions increases (0.02 mol/(m^2^ × h)).

The flux of Ni^2+^ ions through the membrane is 0.04 mol/(m^2^ × h). The difference between the fluxes of Co^2+^ and Ni^2+^ is explained by the fact that NiSO_4_ dissociates in aqueous solutions better than CoSO_4_. The fraction of Ni^2+^ ions in the feed solution is approximately 0.61 (0.39 in the form of its sulfate), while that of Co^2+^ ions is 0.58 (pH = 5.2). The transport number (and hence the flux) of Ni^2+^ ions in pore solution is higher than the transport number of Co^2+^ ions ($t_{Ni^{2+}}$ = 0.07 and $t_{Co^{2+}}$ = 0.06). The $z_{2}D_{2}/z_{1}D_{1}$ ratio for the Li^+^/Ni^2+^ pair is higher than for the Li^+^/Co^2+^ pair (0.76 and 0.71, respectively). This means that it is more difficult to separate Li^+^ and Ni^2+^ ions than Li^+^ and Co^2+^. Along with the measurement error, this probably explains the high flux of these ions through the membrane compared to the experiment with the same parameters without the addition of Ni^2+^ ions. To estimate competing ion fluxes, the change in concentration over time in the chambers of the EBM device is determined against the background of a high concentration of the analyte in the feed solution.

Due to the fact that the fluxes of both Co^2+^ ions and Ni^2+^ ions were positive at 137.5 A/m^2^ (controlled by migration), in order to increase the efficiency of lithium extraction, the current density was reduced to 125 A/m^2^ at the same pressure value (∆*p* = 0.3 bar) (Table 3). This caused the fluxes of cobalt and nickel to become negative, which means that the dominant transport mechanism is convection. However, the lithium flux changed insignificantly, from 0.33 to 0.30 mol/(m^2^ × h). This allowed lithium to be fractionated from the mixed solution. Taking into account that the fluxes of cobalt and nickel ions are negative, the separation coefficient is not available for calculation. The energy consumption ranged from 0.55 to 0.92 kWh/mol Li^+^ for the entire cell (depending on ${\tilde{t}}_{Li^{+}}$), which is comparable with the energy consumption of conventional electrodialysis. The methodology for calculating energy consumption is presented in Ref. [23].

### 3.4. Analysis of Obtained Results

Let us make a brief analysis of the obtained separation characteristics and compare them with similar characteristics found by different authors using other membrane methods. In Table 4, the results from some recent papers on the selective recovery of lithium from leachates of spent LIBs using membrane technologies were compiled. The fluxes of Li^+^ ions through the membrane, as well as the fluxes of competing cations, were calculated using the published data presented in the relevant articles [15,16,17,18,41,42]. Calculations were made using Equations (8) and (9).

It is known that selective electrodialysis (SED) allows separate monovalent and multivalent ions of the same charge sign using special-grade monovalent-ion-selective ion-exchange membranes. In the literature, the examples of the successful application of SED can be found to separate lithium from doubly charged ions of cobalt, nickel, and manganese [15,17,18]. Competing ion fluxes through ion-exchange membranes are determined by the set current and the concentration of ions in the feed solution. The ion separation coefficient $S_{Li^{+}/M^{n+}}$ can reach 5–7 [15,17,18].

Another approach is to use conventional and special-grade ion-exchange membranes together, as well as complexation with EDTA [16,41]. This makes it possible to transfer multiply charged cations, in the form of anionic complexes, through conventional ion-exchange membranes. Lithium does not form a complex and is transported over special-grade membranes. Separation efficiency increases significantly, but the ion separation coefficient cannot always be calculated (is not available), since lithium and doubly charged coexisting ions are transported through different membranes. Only a small portion of doubly charged ions are transported together with singly charged ions, and the ion separation coefficient reaches very high values [16].

In the pressure-driven-membrane method, nanofiltration can be effectively used to separate singly and multiply charged ions. As in the case of electrodialysis, the fluxes of the ions to be separated depend primarily on the magnitude of the driving force (excess pressure) and the concentration of the ions to be separated in the feed solution. The parameters of the selective extraction of lithium from spent LIBs using nanofiltration [42] are close to the parameters obtained using electrodialysis methods (Table 4).

The EBM method can compete with known membrane methods. The flux of lithium through the membrane is comparable to the fluxes of this ion obtained in nanofiltration and electrodialysis. However, the method makes it possible to choose the parameters in such a way that the fluxes of the separated ions are directed in opposite directions (Figure 4). It is possible to choose such conditions when Li^+^ is extracted from chamber I (a positive flux) while the direction of fluxes of competing cations is negative: they cannot pass from chamber I (the feed solution) to chamber II (the receiving solution). Therefore, the selectivity coefficient is theoretically equal to infinity. This opens possibilities for fractionation of the components.

Although the ion-separation coefficient with the EBM method can be much higher than with other membrane methods [1], like other membrane methods, this method is not without drawbacks. The main problem lies in the accuracy of selecting the separation parameters in order to bring the flux of one of the competing ions to zero as accurately as possible or direct it in the opposite direction.

## 4. Conclusions

In this work, the possibility of using the hybrid electrobaromembrane (EBM) method for the separation of Li^+^ and Co^2+^ ions, as well as Li^+^, Co^2+^ and Ni^2+^ contained in leachates of spent LIBs, was studied. For the separation, a track-etched membrane with a pore diameter *d*_av_ of 35 ± 3 nm was used. At this value of *d*_av_, there are no steric hindrances for the ion transfer through the pores. However, the relatively wide pores of track-etched membranes are an important condition for creating oppositely directed convective transport and electromigration. This organization of these two fluxes allows very effective separation of ions with the same charge sign. Based on both the calculations and the results of the EBM separation experiment, the flux of lithium ions through the membrane can be expected to be about 0.3 mol/(m^2^ × h). Under conditions where the feeding and receiving solutions were identical, the flux of cobalt ions was directed from the receiving to the feeding solution and was close to zero (–0.0025 mol/(m^2^ × h)). Formally, through the calculation of the flux ratio, the ion separation coefficient of Li^+^ and Co^2+^ ions was –55. But taking into account that only lithium ions can leave the feed solution, the separation coefficient should be equal to infinity. The presence of coexisting nickel ions in the feed solution leads to a decrease in the separation efficiency of lithium and cobalt if the fluxes of these cations are codirected. However, the separation conditions can be chosen so that only lithium is extracted from the initial solution, while the fluxes of other ions are oppositely directed.

## Figures and Tables

**Table 1:** Some characteristics of ions (at 25 °C) in the feed solution.

Ion	Symbol	Diffusion Coefficient, 10^−9^ m^2^/s [27]	Stokes Radius, Å [28]
Lithium	Li^+^	1.04	2.38
Cobalt	Co^2+^	0.73	3.35
Nickel	Ni^2+^	0.66	2.92
Sulfate	SO_4_^2−^	1.06	2.30

**Table 2:** Characteristics of the TEM#811 track-etched membrane.

Parameter	Value
Thickness	10 μm
Density (dry) *	1.10 ± 0.05 g/cm^3^
Pore density **	5.0 × 10^9^ pores/cm^2^
Pore diameter	35 ± 3.0 nm **
	28 ± 2.0 nm ***
Surface porosity	5.3% ± 1.0%
Water uptake	5%
Hydraulic permeability	0.10 ± 0.02 cm^3^/(cm^2^ × min × bar)
Functional groups	hydroxyl and carboxyl groups [29]
Exchange capacity *	0.064 ± 0.003 mmol/g_wet_
Electrical conductivity (0.1 M NaCl) *	0.81 mS/cm
Integral diffusion permeability coefficient (0.1 M NaCl) *	2.8 × 10^−7^ cm^2^/s

* The results are presented in Ref. [30] for sample #811. ** Estimated by scanning electron microscopy (SEM); *** estimated by hydraulic permeability.

**Table 3:** Comparison of separation efficiency of Li^+^/Co^2+^-ions and Li^+^/Co^2+^/Ni^2+^-ions by EBM method at ∆*p* = 0.3 bar.

Current Density, *i*, A/m^2^	Ions in the Feed Solution	$c_{M^{n+}}^{0},\;g/L$	$j_{M^{n+}},\;\mathrm{mol}/(m^{2}\times \;h)$	$S_{Li^{+}/M^{n+}}$
Li^+^/Co^2+^-containing feed solution				
137.5	Li^+^	0.69	0.28	–
	Co^2+^	2.95	–0.0025	n/a
125	Li^+^	0.69	–0.29	–
	Co^2+^	2.95	–0.022	8
Li^+^/Co^2+^/Ni^2+^-containing feed solution				
137.5	Li^+^	0.69	0.33	–
	Co^2+^	1.47	0.02	4
	Ni^2+^	1.47	0.04	2
125	Li^+^	0.69	0.30	–
	Co^2+^	1.47	–0.02	n/a
	Ni^2+^	1.47	–0.005	n/a

**Table 4:** Comparison of recovery/rejection of used lithium using membrane methods.

Method	Membrane	Feed Solution	Experiment Details	$j_{Li^{+}},\;\mathrm{mol}/(m^{2}\times \;h)$	Competing Cation, C^+^	$j_{M^{n+}},\;\mathrm{mol}/(m^{2}\times \;h)$	$S_{Li^{+}/M^{n+}}$
Selective electrodialysis Ref. [18]	Cell with monovalent selective Selemion CSO (Asahi Glass, Tokyo, Japan) or Neosepta CIMS membranes, as well as Neosepta AMX (Astom, Shunan, Japan)	Leach solution of NMC111 cathodic materials: 2.60 g/L Li^+^, 7.88 g/L Co^2+^, 8.01 g/L Ni^2+^, 4.40 g/L Mn^2+^, 51.45 g/L SO_4_^2–^ (pH = 2.8)	125 A/m^2^	1.92 over the Selemion CSO	Co^2+^	0.56	1.25
					Ni^2+^	0.54	1.3
					Mn^2+^	0.29	1.4
				3.05 over the Neosepta CIMS	Co^2+^	0.20	5.6
					Ni^2+^	0.18	6.1
					Mn^2+^	0.12	5.4
Selective electrodialysis Ref. [17]	5 cell pair with monovalent selective PC-MVK & PC-MVA membranes (PCA GmbH, Heusweiler, Germany)	0.1 g/L Li^+^& 0.3 g/L Co^2+^	5 V (1 V/cell) ~15 A/m^2^	0.1	Co^2+^	8.8 × 10^−3^	4
Selective electrodialysis Ref. [15]	Cell with laboratory-made selective PAN-5C8Q membrane as well as Neosepta ASE (Astom, Shunan, Japan)	0.027 g/L Li^+^, 0.108 g/L Co^2+^, 0.049 g/L Ni^2+^	5 V	0.047	Co^2+^	0.044	0.5
					Ni^2+^	1.5 × 10^−3^	7
Conventional electrodialysis + complexation & selective electrodialysis Ref. [16]	Cell with monovalent selective Neosepta CMS membrane, as well as Neosepta AMX, Neosepta CMX (Astom, Japan) and PCA PC 400D (PCA GmbH, Heusweiler, Germany)	Leach solution of NMC111 cathodic materials: 0.07 g/L Li^+^, 0.2 g/L Co^2+^, 0.2 g/L Ni^2+^, 0.18 g/L Mn^2+^ & SO_4_^2–^ (pH ~ 1.5)	18 V (Stage 1)	0.165 over the Neosepta CMS (Stage 3)	Ni-EDTA^n–^ over the PCA PC 400D (Stage 1)	0.057	n/a
			18 V (Stage 2)		Co-EDTA^n–^ over the PCA PC 400D (Stage 2)	0.042	n/a
			3 V (Stage 3)		Mn^2+^ over the Neosepta CMS (Stage 3)	5.7 × 10^−4^	92
Bipolar membrane electrodialysis + complexation Ref. [41]	Cell with Neosepta BP-1E bipolar membrane (Astom, Japan), as well as Selemion CMV and Selemion AMV (Asahi Glass, Tokyo, Japan)	0.14 g/L Li^+^, 1.18 g/L Co^2+^, 3.72 g/L NO_3_^–^, 1.84 g/L Na^+^ (pH = 7.0)	20 V	0.77 over the Selemion CMV	Co-EDTA^n–^ over the Selemion AMV	0.33	n/a
Nanofiltration Ref. [42]	Ccell with VNF2 nanofiltration membrane (Vontron Membrane Technology Ltd., Beijing, China)	Leach liquor of lithium-iron-phosphate spent LiBs: 23.9 g/L Li^+^, 0.78 g/L Ni^2+^, 0.58 g/L Co^2+^, 0.67 g/L Mn^2+^, 27.3 g/L Fe^3+^, 0.18 g/L Al^3+^, 0.28 g/L Cr^3+^, 0.059 g/L Cu^2+^, 11.0 g/L PO_4_^3–^ (pH = 2.2)	10 bar	0.67	Co^2+^	3.0 × 10^−4^	6.2
					Ni^2+^	5.7 × 10^−4^	4.7
					Mn^2+^	3.0 × 10^−4^	8.2
Hybrid electro-baromembrane (EBM) method [this study]	Cell with TEM #811 track-etched membrane, as well as two MA-41 (JCC Shchekinoazot, Pervomayskiy, Russia)	0.69 g/L Li^+^, 2.95 g/L Co^2+^, 9.6 g/L SO_4_^2–^ (pH = 4.2–4.4) or 0.69 g/L Li^+^, 1.47 g/L Co^2+^, 1.47 g/L Ni^2+^, 9.6 g/L SO_4_^2–^ (pH = 5.2)	137.5 A/m^2^ 0.3 bar	0.36 *	Co^2+^	0.01 *	18 *
				0.33	Co^2+^	0.02	4
					Ni^2+^	0.04	2

* Results of one of the experiments with codirectional fluxes of separated ions at given parameters, in contrast to the results discussed above (Figure 4, Table 3), where the average values for four series of experiments are presented.

## Data Availability

Not applicable.
